# New Method for Detecting Flange Fracture Initiation in Incremental Radial Extrusion

**DOI:** 10.3390/ma17051054

**Published:** 2024-02-25

**Authors:** Grzegorz Winiarski

**Affiliations:** Faculty of Mechanical Engineering, Lublin University of Technology, 38 D Nadbystrzycka Str., 20-618 Lublin, Poland; g.winiarski@pollub.pl

**Keywords:** fracture, incremental radial extrusion, flange forming, FEM

## Abstract

This study investigates flange fracture formation in unconventional incremental radial extrusion. This manufacturing technique involves using rings with a gradually increasing inside diameter for constraining the free flow of material in the radial direction. As a result, the shaped flange has a constant thickness and a significantly larger diameter than that formed using the standard extrusion process conducted without the use of rings. EN AW 6060 aluminum alloy tube sections were used as the billet material, and the extrusion process was conducted under cold forming conditions at ambient temperature. For the determination of material fracture initiation, a new method was proposed involving the analysis of strain, strain rate and values of the normalized Cockcroft–Latham fracture criterion integral. The main advantage of the new method is that it allows for the prediction of fracture initiation via only FEM results analysis, i.e., it is not necessary to carry out additional experiments aimed at calibrating or determining limit parameters of a given material. It was shown that the occurrence of differences in the distribution of the above-mentioned parameters coincided with flange fracture initiation.

## 1. Introduction

Extrusion is a technique that enables the manufacturing of a wide range of products such as rods, structural profiles, and forgings with complex geometries. The conventional techniques of forward, backward, and radial extrusion are constantly being modified to improve process conditions and extruded-part quality. Matsumoto et al. [1] investigated the combined forward–backward extrusion process in which the punch performed a pulsating motion. A hole was made in the axis of symmetry of the punch for liquid lubricant supply into the contact zone between the punch nose and the material. The results showed that the implementation of the extrusion process with pulse punch motion and the periodic supply of lubricant led to a significant reduction in the frictional forces. A similar problem was raised by Maeno et al. [2], who investigated the backward extrusion process with pulsating punch motion. The pulsating motion generated negative pressure under the punch bottom, causing the gaps formed in the punch grooves to be filled with lubricant. It was shown that the maximum force was lower than in conventional backward extrusion and that the tool wear was reduced. Lowrie et al. [3] studied the backward extrusion process in which punch ejection load could be eliminated or reduced. To that end, they used an insert attached to the punch nose. The insert made the punch deform elastically and increased its diameter. When the load was removed, the diameter decreased, as a result of which clearance occurred between the extruded hole and the tool, and the load of the workpiece on the punch was reduced. As a result, the load required to eject the punch was significantly reduced and the product quality improved. Fatemi-Varzaneh et al. [4] and Faraji et al. [5] investigated the accumulative back extrusion (ABE) process for the fabrication of bulk ultra-fine-structured materials. This process involved forming a sleeve with a bottom using backward extrusion first and then re-deforming the sleeve to the starting material form, i.e., a tube. The results showed that the distribution of strains after one deformation cycle was inhomogeneous, but after two cycles the parameter significantly improved. Also, the strains showed a higher homogeneity when the punch diameter was increased. Fine-grain structure parts can also be produced using backward extrusion with a rotating tool. The rotating motion can be performed either with the die [6,7] or with the punch [8,9]. As a result, the strains are three times higher and the force is decreased by 20–30% compared to those in the conventional extrusion method. In addition to that, the technique leads to a several-fold reduction in grain size and increased product microhardness. The key parameters in this process are the tool speed and the geometry of the punch and container. A similar process was investigated by Jafarzadeh et al. [10]. In a new process called friction stir radial backward extrusion (FSRBE), material is extruded in the radial and backward direction. Additionally, the die performs a rotary motion. Studies have shown that the use of this technique makes it possible to reduce the grain size by more than six times as well as to increase the tensile strength, elongation, and microhardness of the deformed material. Hollow parts can be produced using modified forward extrusion methods [11,12,13]. The material flows through a variable geometry die cavity that is created between the mandrel and the die. As a result, considerable strains occur, leading to dynamic recrystallization and structure fragmentation. Flanges on hollow parts are usually formed from tubes through radial extrusion. A significant failure mode in this method is the uneven thickness of a shaped flange and thus the risk of flange fracture. To eliminate the above, extrusion is conducted with the use of an additional ring in the zone where the material flow direction is changed [14] or the free flow of material in the radial direction is constrained by means of a deformable ring [15].

Performing a numerical analysis does not pose major problems in terms of product geometry and force parameters, the determination of material fracture initiation in the extrusion processes is a more complex issue. Ko et al. [16] used the Cockcroft–Latham criterion to analyze cracks in the central part of a part produced using forward extrusion. The limit value of the criterion was determined using the Bridgman method. The results showed that the material fracture was more likely to occur if the cross-sectional reduction of the billet was increased and a higher working angle of the die was used. Similar studies were conducted by Saanouni et al. [17] and Soyarslan et al. [18], who applied the continuum damage mechanics (CDM) theory. Soyarslan et al. [19] used the CDM theory to analyze chevron cracking in forward extrusion with counter pressure. It was shown that the use of counter pressure prevented material fracture initiation and that the use of appropriate pressure values made it possible to completely eliminate that defect in the extruded part. Choi et al. [20] used the specific plastic work criterion and the Cockcroft–Latham criterion to study material fracture in extrusion processes, determining the limit value of these criteria under instability and tensile fracture conditions. The formation of external cracks was predicted using the first criterion, while internal cracking was predicted using the other criterion. Balasundar et al. [21] investigated the repetitive upsetting–extrusion process (one of many variations of the severe plastic deformation process), showing that internal defects could be prevented using a modified die design. To investigate chevron cracking in a multi-step forward process, Sebek et al. [22] applied the calibration criteria developed by Bai and Wierzbicki, Mohr-Coulomb, and Lou et al. in the new extremely low-stress triaxiality fracture test. It was shown that the first criterion correctly indicated the initiation of fracture in the sixth step of the process, but the predicted number and shape of the cracks were incorrect. The second criterion predicted that fracture would occur in the fifth step, while the third criterion indicated the onset of cracking in step four, whereas in reality it occurred in the sixth step of the process. Terhorst et al. [23] used the temperature-dependent limit value of the normalized Cockcroft–Latham fracture criterion to improve the accuracy of numerical modelling of the extrusion process. In effect, it was possible to predict the initiation and location of material fracture under both cold and hot forming conditions. Wang et al. [24] analyzed the surface cracking of an Al-Li alloy extrusion profile. It was found that material fracture took place when the temperature of the near-surface layers of the profile was too high and the tensile plastic work accumulation exceeded the critical value. For the numerical prediction of material fracture, a new criterion was developed taking into account the effects of temperature and strain rate. The critical value of the criterion is determined using an appropriate calibration test. Kubik et al. [25] used the uncoupled ductile fracture criteria to investigate cracking in the six-pass cold forward extrusion process. Experiments, including the new small punch test, were conducted to calibrate the criteria. The results showed that different criteria perform differently, predicting fracture too early (Ganjiani criterion), no fracture (KHPS2 criterion), or according to the experimental results (DF2021 criterion). Park [26] showed that the radial extrusion process was prone to the following failure modes: flange fracture, waviness, and non-uniform thickness. To resolve these problems, the bottom surface of the container had a conical design (described with an inclination angle of 0.7°). In effect, the stress state in the flange changed and the tensile circumferential stresses decreased. The use of the normalized Cockcroft–Latham criterion showed that the solution significantly reduced the value of the integral (allowing for a larger diameter flange to be formed) compared to the container with its bottom surface perpendicular to the axis of the workpiece. The accuracy of fracture criteria primarily depends on the knowledge of their critical values. The problem of determining these values was studied by Wierzbicki et al. [27], who calibrated fracture criteria such as constant equivalent strains, fracture-forming limit diagram, maximum shear stress, as well as the fracture criteria developed by Johnson-Cook, Xue-Wierzbicki, Wilkins, CrachFEM, and Cockcroft–Latham. Cristino et al. [28] developed a new method for determining the limit values of the criteria based on circle grid analysis and plasticity theory, which was dedicated to flange formation using single-point incremental forming. Kubik et al. [29] calibrated the four uncoupled criteria using a new cylindrical test specimen with an undercut, the use of which resulted in very-low-stress triaxiality values. Pater et al. [30] also proposed a new method for fracture criteria calibration involving performing a rotary compression test with the use of cylindrical test specimens.

The literature review has shown that extrusion techniques are constantly being developed. New forming methods are being developed to reduce failure modes in already existing processes. Another trend is to develop testing tools that are compatible with the new techniques. Regarding material fracture in extrusion processes, it can be observed that the prediction of fracture initiation is a complex issue. The use of existing failure criteria is related to the determination of their limit values that are determined via calibration tests. The best results are obtained when the stress and strain in the material fracture zone in the calibration test reflect those occurring in the real process. Moreover, in certain cases, the selected fracture criteria may indicate the location and the initiation of material fractures inaccurately. For this reason, alternative methods are sought to determine the initiation of material fracture already at the stage of manufacturing technique design. 

The objective of this study is to investigate a new method for predicting material fracture initiation in radial extrusion processes. The main assumption of the proposed method is to determine the initiation of material fracture based on only FEM results’ analysis, i.e., without conducting additional experiments aimed at, e.g., calibrating or determining limit parameters. This study investigates the phenomenon of fracture in a flange formed using unconventional incremental radial extrusion. A numerical model of the process is developed to perform a detailed analysis of strains and values of one of the most widely used failure criteria. As a result, the initiation of material fracture in the flange is determined, and the obtained numerical results are then compared with experimental findings.

## 2. Materials and Methods

**Figure 1:**
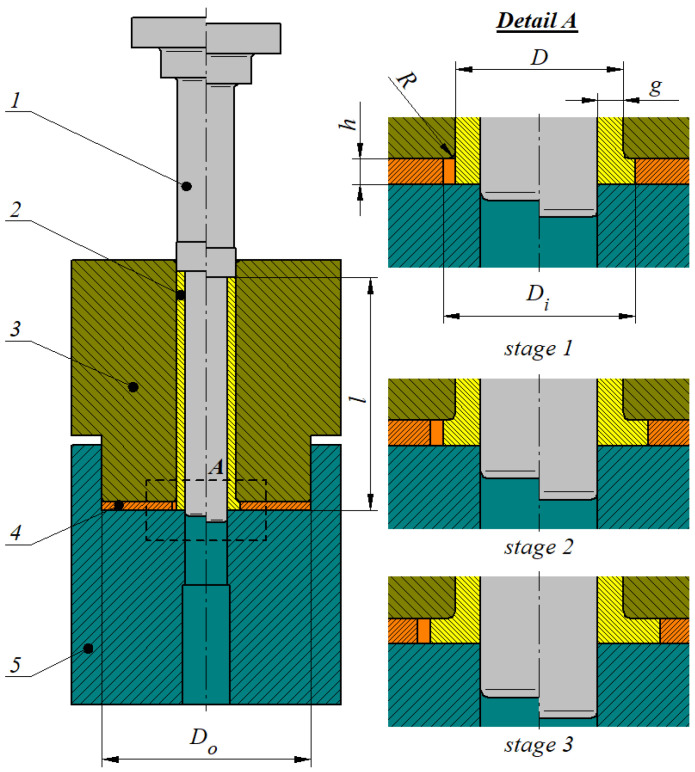
Schematic design of incremental radial extrusion of a flange: 1—punch, 2—billet, 3—container, 4—ring, 5—base.

A schematic design of the analyzed unconventional incremental radial-extrusion process for a flange in hollow parts is shown in Figure 1. As a result of the load exerted by a punch 1 on a billet 2, a closed die cavity formed between a base 5, container 3, ring 4, and punch 1 is filled with the deformed material. The first stage of shaping the flange ends when the closed die cavity is completely filled. Successive stages of the extrusion process proceed in the same way and are conducted using rings of an increasing inside diameter D_i_, which makes it possible to shape a flange with an increasing diameter but a constant thickness h.

Numerical calculations of the process were conducted in DEFORM-3D v11 software. They were performed using the finite element method according to the parameters shown in Table 1. The workpiece was modeled as a rigid plastic object. Three-dimensional, tetragonal, four-node elements were used to discretize it. The flow curve of the deformed material, i.e., EW AW 6060 aluminum alloy, was described using a constitutive equation, while the other parameters of the material model were taken from the program database. Tools were modeled as rigid objects. Contact conditions with the workpiece were described using a shear friction model with consideration of heat transfer between objects. The process was defined as fully three-dimensional. It means that the numerical model did not use any simplifications in the form of assuming, for example, an axisymmetric state of strain.

The results of the author’s previous study [31] were used to conduct further investigation into the process, focusing on flange fracture. For this purpose, the distribution of effective strain (Equation (1)), strain rate (Equation (2)), and values of the normalized Cockcroft–Latham fracture criterion integral (Equation (3)) were presented.

(1) $$\varepsilon =\frac{\sqrt{2}}{3}\cdot \sqrt{{\left(\varepsilon_{1}-\varepsilon_{2}\right)}^{2}+{\left(\varepsilon_{2}-\varepsilon_{3}\right)}^{2}{+\left(\varepsilon_{3}-\varepsilon_{1}\right)}^{2}}$$

(2) $$\dot{\varepsilon }=\frac{d\varepsilon }{dt}$$

(3) $$C=\int_{0}^{\varepsilon }\frac{\sigma_{1}}{\sigma_{i}}d\varepsilon$$

$$\mathrm{where}\;\sigma_{i}=\frac{\sqrt{2}}{2}\cdot \sqrt{{\left(\sigma_{1}-\sigma_{2}\right)}^{2}+{\left(\sigma_{2}-\sigma_{3}\right)}^{2}{+\left(\sigma_{3}-\sigma_{1}\right)}^{2}}$$

where

*ε*—effective strain;
*ε*_1_, *ε*_2_, *ε*_3_—principal strains;
$\dot{\varepsilon }$—effective strain rate;
*C*—value of the integral according to the normalized Cockcroft-Latham fracture criterion;
*σ*_1_, *σ*_2_, *σ_3_*—principal stresses;
*σ_i_*—effective stress.

**Figure 2:**
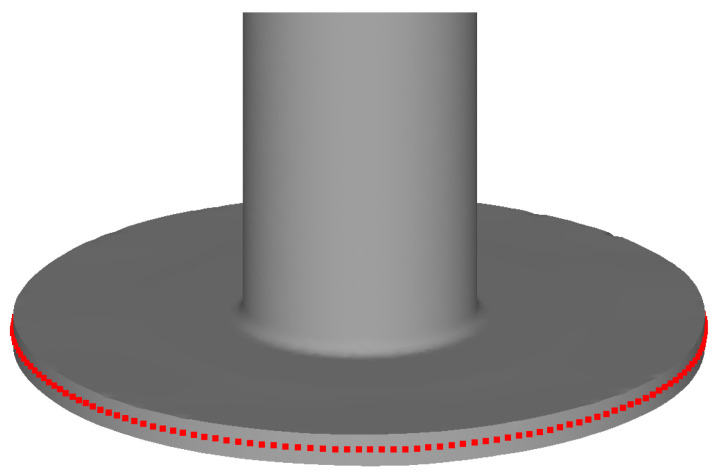
Location of the sensors on an extruded part.

In addition to the maps showing the distribution of the aforementioned parameters, their values recoded using 180 sensors were analyzed. The values in the sensors were extracted with the post-processing of the numerical model. At the beginning of the process, the sensors were spaced evenly (every 2°) on the outer cylindrical surface of the billet, at a distance from the base equal to half the thickness h of the ring. The sensors moved together with the workpiece (Figure 2) and were located in accordance with their numbering, i.e., Sensor 1 was located between Sensors 180 and 2, Sensor 2 was located between Sensors 1 and 3, and so on. Based on the values of the parameters recorded with the sensors, the following indicators were defined:

- Stress triaxiality

(4) $$\eta =\frac{\sigma_{m}}{\sigma_{i}}$$

$$\mathrm{where}\;\sigma_{m}=\frac{\sigma_{1}+\sigma_{2}+\sigma_{3}}{3}$$

- Ratio between the maximum and the minimum effective strain at a given moment of time in all sensors:

(5) $${\delta \varepsilon }_{i}=\frac{max\left\{\varepsilon_{i-s1},{\;\varepsilon }_{i-s2},\;{\;\varepsilon }_{i-s3},\;\ldots ,\;\varepsilon_{i-s180}\right\}}{min\left\{\varepsilon_{i-s1},{\;\varepsilon }_{i-s2},\;{\;\varepsilon }_{i-s3},\;\ldots ,\;\varepsilon_{i-s180}\right\}}$$

- Increase in the effective strain value in individual sensors:

(6) $${∆\varepsilon }_{i,sj}=\frac{\varepsilon_{i+1,sj}-\varepsilon_{i,\;sj}}{\varepsilon_{i,sj}}\cdot 100\%$$

- Ratio between the maximum and the minimum value of the normalized Cockcroft–Latham fracture criterion integral at a given moment of time in all sensors:

(7) $${\delta C}_{i}=\frac{max\left\{C_{i-s1},C_{i-s2},C_{i-s3},\ldots ,C_{i-s180}\right\}}{min\left\{C_{i-s1},C_{i-s2},C_{i-s3},\ldots ,C_{i-s180}\right\}}$$

- Increase in the value of the normalized Cockcroft–Latham fracture criterion integral in individual sensors:

(8) $${∆C}_{i,sj}=\frac{C_{i+1,sj}-C_{i,\;sj}}{C_{i,sj}}\cdot 100\%$$

where

*σ_m_*—mean stress;
*ε_i−s_*_1_, *ε_i−s_*_2_, *ε_i−s_*_3_, *…*, *ε_i−s_*_180_—effective strain at the i-th moment of time in sensors s1, s2, s3, …, s180;
*ε_i,sj_*—effective strain at the i-th moment of time in the j-th sensor;
*C_i−s_*_1_, *C_i−s_*_2_, *C_i−s_*_3_, *…*, *C_i−s_*_180_—values of the normalized Cockcroft-Latham fracture criterion integral at the i-th moment of time in sensors s1, s2, s3, …, s180;
*C_i,sj_*—values of the the normalized Cockcroft–Latham fracture criterion integral at the i-th moment of time in the j-th sensor.

The initiation of the flange fracture was predicted by analyzing the variations in the values of effective strain, strain rate, and the normalized Cockcroft–Latham fracture criterion integral. It was assumed that any noticeable changes and scatter in these values on the flange edge would indicate the inhomogeneity of the stresses and strains, which—in turn—would indicate the loss of axial symmetry of the process and the formation of zones with varying material effort. The above-mentioned phenomena occur at the moment of material fracture, which was confirmed with the experimental results. The procedure for predicting flange fracture initiation involves the following steps:

1. FEM analysis of the incremental radial flange extrusion process;
2. Determination of the distribution of effective strains, strain rates, and values of the normalized Cockcroft–Latham fracture criterion integral in extruded parts;
3. Determination of parameters describing the scatter and increment of effective strains, strain rates and normalized Cockcroft–Latham fracture criterion integral values on the flange edge;
4. Determination of the moment of change in the values of the above parameters;
5. Determination of the maximum flange diameter.

## 3. Results

**Figure 3:**
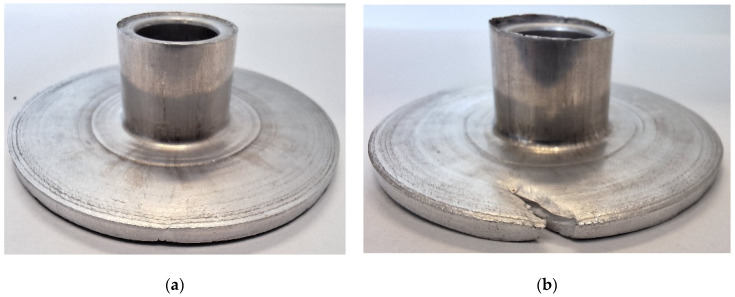
Hollow part with a flange: (**a**) right before fracture initiation, (**b**) with visible fracture.

**Figure 4:**
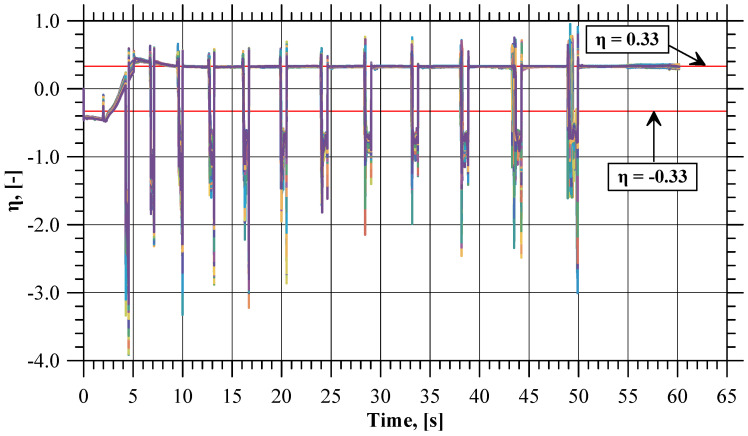
Stress triaxiality values recorded with the sensors during flange incremental radial extrusion process.

The experimental results of the incremental radial extrusion of hollow parts have shown that the limit value of the flange diameter at which fracture occurs is 59 mm. The material fracture occurs on the flange edge and is preceded by the occurrence of a local decrease in material thickness (Figure 3). The use of rings for constraining the free flow of material in the radial direction makes it possible to shape a flange, having constant thickness and 30% larger diameter than that obtained using the extrusion process conducted without the rings [31]. The fact that a relatively large flange diameter can be obtained with the use of the rings results from a cyclic, favorable change in the state of stress occurring in the flange. In the stress triaxiality course shown in Figure 4 for all 180 sensors, a characteristic pattern of variation can be observed. Local and repeated minimum values indicate the appearance of contact between the material and the ring of a given inside diameter. Then, the stress triaxiality value is well below −0.33. This stress value is safe for the material, because it does not lead to the formation of cracks. When the ring is changed to have a larger inside diameter, the flange lateral surface is not in contact with the tool. Then, in the flange zone, the flow of material in the radial direction is free, which is observed using the stress triaxiality value oscillating around 0.33. At such a stress state, a flange fracture may be due to a shear fracture or ductile fracture. Consequently, incremental forming makes it possible to change cyclically the stress state in the flange, which consequently delays the moment of fracture.

**Figure 5:**
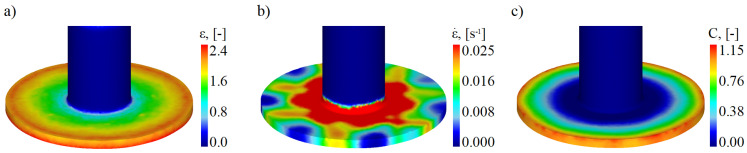
Distributions of (**a**) effective strain, (**b**) strain rate, (**c**) values of the normalized Cockcroft–Latham fracture criterion integral in a hollow part, at the moment of reaching the limit (due to fracture) flange diameter of 59 mm.

Maps of the distribution of effective strain, strain rate, and values of the normalized Cockcroft–Latham fracture criterion integral in a hollow part, having a flange with the limit diameter of 59 mm, are shown in Figure 5. For all maps, the scale was adjusted so as to present as clearly as possible the distributions of values on the flange edge where the fracture initiates. Regarding the distribution of effective strain and damage function, the maximum values are located on the flange edge and decrease with decreasing the distance from the axis of symmetry of the workpiece. It can be observed that the values of the two afore-mentioned parameters are not the same around the flange edge. This relationship is much more evident for the strain rate distribution. Zones can be distinguished where the values of all analyzed parameters reach local maximum and minimum values. It means that the state of stress and strain occurring on the flange edge is not homogeneous. This is due to the fact that the fracture of the material occurs only at a single point on the circumference of the flange, as confirmed with experimental studies. Thus, the process cannot be considered as axisymmetric, and the appearance of an uneven distribution of the studied parameters describing the state of strain and stress proves that the applied numerical model is in agreement with the real process.

## 4. Discussion

### 4.1. Effective Strain

**Figure 6:**
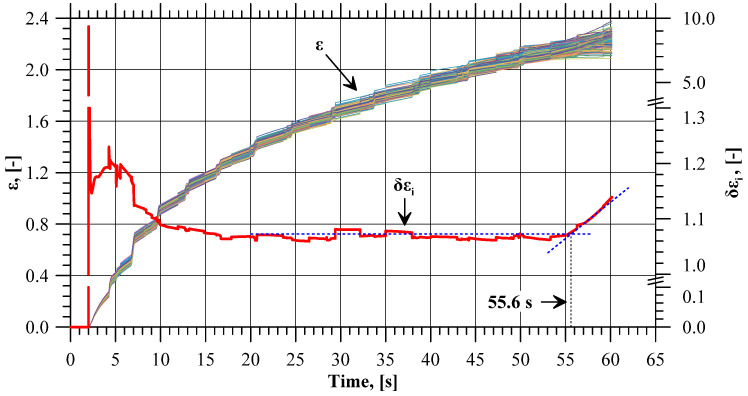
Values of effective strain (*ε*) recorded with the sensors and the ratio between maximum and minimum effective strain values (*δε_i_*) with the trend lines marked in blue.

**Figure 7:**
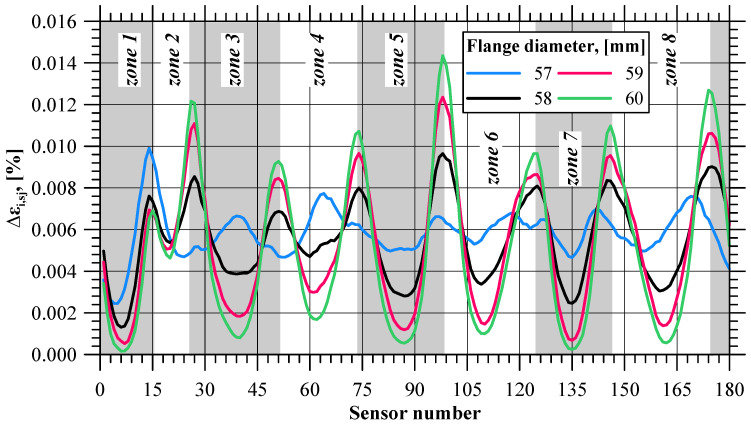
Effective strain increase (∆*ε_i,sj_*) at selected stages of the extrusion process.

**Figure 8:**
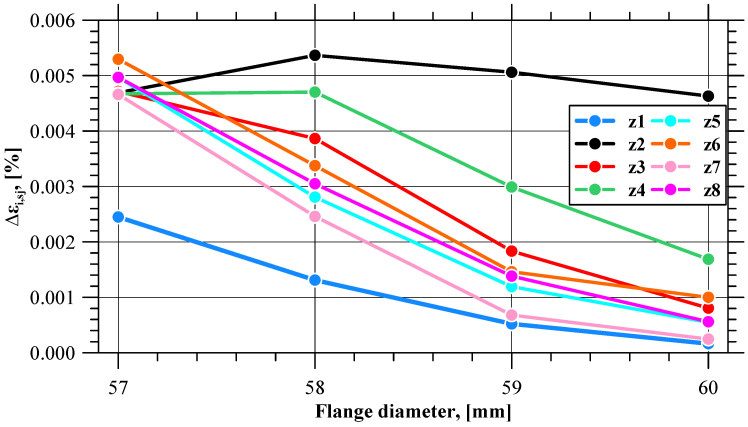
Minimum values of effective strain increase (∆*ε_i,sj_*) in selected zones on the flange.

Curves illustrating the pattern of effective strain value variations in all 180 sensors are shown in Figure 6. Qualitatively, the curves are similar. The effective strain value on the flange edge increases with the progress of the process. In terms of quantity, however, the values clearly differ, especially at the final stage of the process, which indicates a non-uniform distribution of this parameter. The effective strain non-uniformity is expressed by means of the *δε_i_* indicator. At the initial stage of the extrusion process (until 10s), the tube is shaped into a flange, and hence the *δε_i_* value is slightly higher than in the successive, stabilized stage of the extrusion process. In the stable stage of the process, the value of this indicator is almost constant and oscillates around 1.07. The trend lines of the *δε_i_* indicator for the stable and final phases of the extrusion process indicate that after exceeding 55.6 s of the process duration, the *δε_i_* value clearly increases, which shows that the difference between the maximum and the minimum value of effective strain is becoming larger. At this point, the flange has a diameter of 59.16 mm, which is very close to the limit value of 59 mm. Figure 7 shows the values of the ∆*ε_i,sj_* indicator describing the increase in the effective strain value in individual sensors at selected stages of the extrusion process (near the moment when the *δε_i_* value increases), i.e., when the flange diameter is 57, 58, 59, and 60 mm. The patterns of all curves are sinusoidal, which indicates an uneven increase in the effective strain on the circumference of the flange. One can distinguish eight zones in which the local minimum values of the ∆*ε_i,sj_* indicator are located. The minimum values from each zone are shown in Figure 8 as a function of the flange diameter. In five zones, i.e., z1, z5, z6, z7, z8, the minimum values of the ∆*ε_i,sj_* indicator are similar. For the flange diameters of 57 to 59 mm, the ∆*ε_i,sj_* value decreases at a constant rate, while for the flange diameters larger than 59 mm (which is the limit value due to fracture), the rate of decrease in the ∆*ε_i,sj_* value is lower.

An analysis of the effective strain distribution on the flange edge reveals that the indicators *δε_i_* and ∆*ε_i,sj_* indicate a non-uniform distribution of this parameter. Immediately before the flange fracture initiation, one can observe the formation of dead zones in which the effective strain value is slightly increased, as well as of zones with significantly higher material effort wherein the first crack occurs. In addition to that, there is a simultaneous change in the values of the parameters *δε_i_* and ∆*ε_i,sj_* when the flange reaches the limit diameter value.

### 4.2. Strain Rate

**Figure 9:**
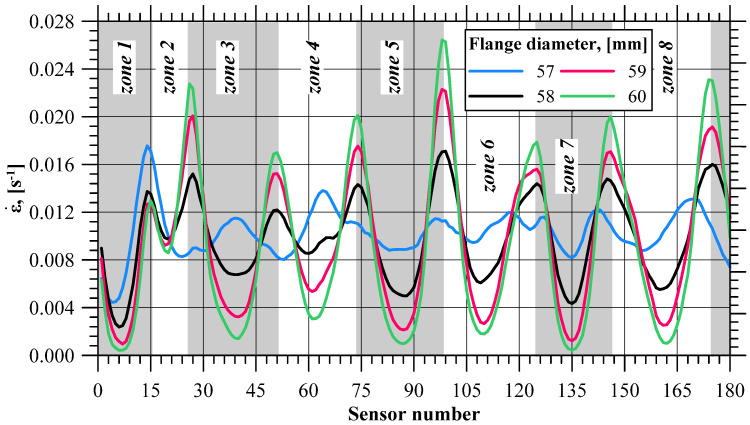
Strain rate $\dot{\left(\varepsilon \right)}$ recorded with sensors.

Figure 9 shows the strain rate values recorded with the sensors at selected stages of the extrusion process (near the moment when the *δεi* value increases). As in the case of the effective strain increase ∆*ε_i,sj_*, the strain rate values on the flange edge are not the same. One can distinguish zones where the material flow rate reaches the local maximum and minimum values. In addition to that, these zones coincide with the five zones distinguished in the effective strain analysis. As the flange diameter increased, more and more differences are visible between the minimum and maximum strain rates recoded with the sensors. This means that dead zones are formed on the flange edge where the material is deformed to a lesser and lesser degree. On the other hand, in-between these zones there occur zones of increased material effort where the fracture initiates.

**Figure 10:**
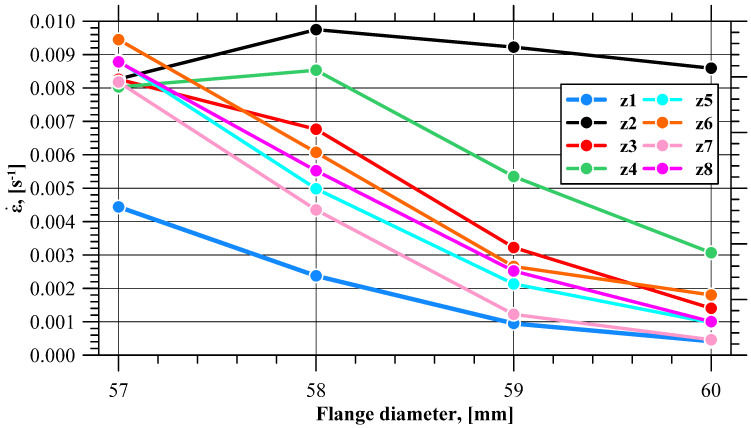
Minimum strain rate $\dot{\left(\varepsilon \right)}$ in selected zones of the flange.

An analysis of the strain rates recorded with the sensors demonstrates that there is a noticeable non-linear decrease in the strain rate when the fracture initiates. Based on the minimum strain rate values in the zones on the flange edge shown in Figure 10, it can be observed that once the actual flange fracture initiates, there are five zones (like in the case of effective strain) where the strain rate decreases at a lower rate than before, reaching the limit flange diameter value.

### 4.3. Cockcroft–Latham Integral

**Figure 11:**
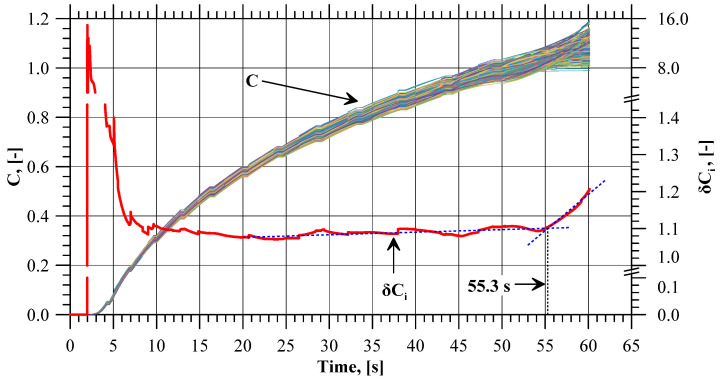
Values of the Cockcroft–Latham integral (*C*) recorded with the sensors and the ratio between maximum and minimum values of the Cockcroft–Latham integral (*δC_i_*) with the trend lines marked in blue.

**Figure 12:**
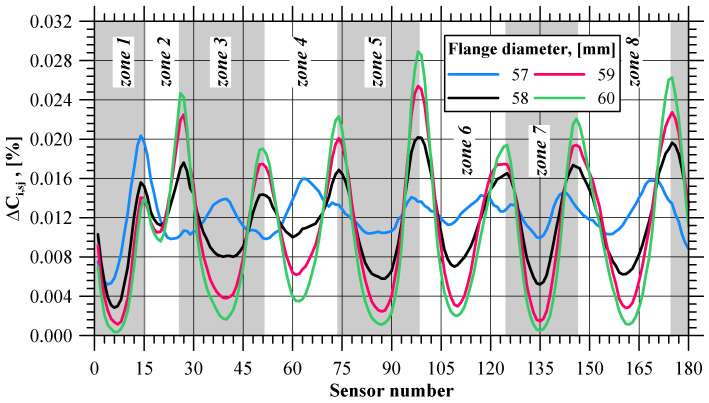
Increase in the Cockcroft–Latham integral value (∆*C_i,sj_*) at selected stages of the extrusion process.

**Figure 13:**
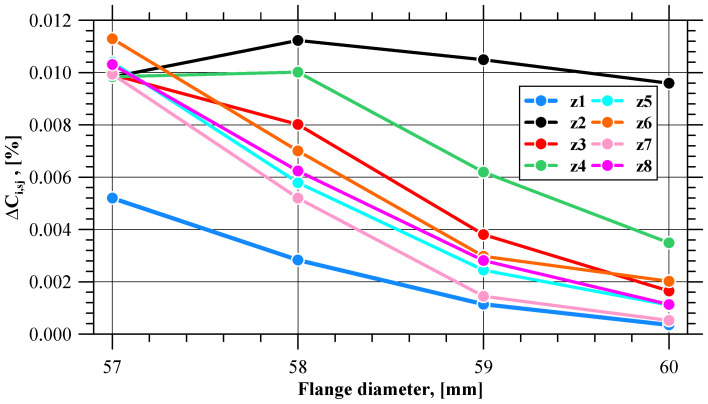
Minimum values of the Cockcroft–Latham integral increase (∆*C_i,sj_*) in selected zones of the flange.

For the determination of flange fracture initiation, the values of the normalized Cockcroft–Latham ductile fracture criterion were also analyzed. Figure 11 shows the integral values recorded with all the sensors. The curve patterns are similar to those obtained for effective strain. Toward the end of the extrusion process, the scatter between the maximum values for a given moment becomes more and more noticeable. The *δC_i_* value increases after 55.3 s of the process duration, i.e., after the flange reaches a diameter of 59.05 mm (which is almost the limit value). Thus, it is not necessary to know the critical value of the integral for the analyzed case in order to determine the initiation of material fracture. This is also confirmed using the integral C value increase observed at the selected stages of the extrusion process (near flange fracture initiation) shown in Figure 12. Like in the strain and strain rate analysis, it can be observed that at the final stage of the extrusion process, zones are formed where the integral increase values reach the local extremes. Moreover, these are the same zones as in the case of strain distribution and strain rate. In addition to that, in these zones, the minimum values of the integral increase (Figure 13) show the same trend as was observed for the two previously analyzed parameters, clearly indicating the flange fracture initiation.

## 5. Conclusions

The results of the study investigating the incremental radial extrusion of flanges in hollow parts lead to the following conclusions:

- Incremental radial extrusion makes it possible to form flanges on hollow parts, these flanges having constant thickness and a relatively large outside diameter;
- The use of rings with an increasing inside diameter for constraining the free flow of material in the radial direction causes a cyclic change in the state of strain and stress in the flange, which has a positive effect on the quality of the extruded parts;
- When material fracture initiates, the values of effective strain, strain rate, and the normalized Cockcroft–Latham fracture criterion integral on the flange edge begin to differ from those in other zones of the flange;
- The identification of the moments of changes in the values of the above-mentioned parameters, and thus the determination of flange fracture initiation, is possible via only numerical results analysis, which means that the proposed method for a limited flange diameter calculation does not require the performance of additional experiments aimed at calibrating or determining the limit parameters of the extruded material.

## Figures and Tables

**Table 1:** Experimental conditions of incremental radial extrusion process for producing a flange.

Name of the Parameter	Parameter Value
Billet material	aluminum alloy EW AW 6060 in annealed state
Billet dimensions	D = 20 mm, g = 3 mm, l = 80 mm
Container working edge rounding radius	R = 1 mm
Ring dimensions	D_i_ = 23, 26, 29, 32, 35, 38, 41, 44, 47, 50, 53, 56 mm, D_o_ = 70 mm, h = 3 mm
FEM software	Deform-3D v11
Flow curve equation for tested aluminum alloy	*σ_p_ = 147.5·ε^0.2^* where *σ_p_*—plastic stress, *ε*—strain
Initial temperature of billet and tools	20 °C
Coefficient of heat transfer between workpiece and tools and between workpiece and environment	12 kW/m^2^K, 0.02 kW/m^2^K
Friction factor between workpiece and tools	0.2
Punch velocity	50 mm/min
Testing machine used in experiments	Instron 1000HDX

## Data Availability

Data are contained within the article.
